# Differential HbA1c response in the placebo arm of DPP-4 inhibitor clinical trials conducted in China compared to other countries: a systematic review and meta-analysis

**DOI:** 10.1186/s40360-016-0084-7

**Published:** 2016-09-07

**Authors:** Lingyu He, Shu Liu, Chun Shan, Yingmei Tu, Zhengqing Li, Xiaohua Douglas Zhang

**Affiliations:** 1 Research School of Finance, Actuarial Studies & Statistics, The Australian National University, Canberra, ACT 2601 Australia; 2 Clinical Research, MSD China R&D Center, Beijing, 100015 China; 3 National Institutes for Food and Drug Control, Beijing, 100050 China; 4 MSD China R&D Center, Beijing, 100015 China; 5 Faculty of Health Sciences, University of Macau, Taipa, Macau

**Keywords:** Dipeptidyl peptidase-4 inhibitor, Diabetes mellitus, T2DM, Differential HbA1c response, Meta-analysis

## Abstract

**Background:**

It has been observed that the efficacy of dipeptidyl peptidase-4 (DPP-4) inhibitors as compared to the placebo groups in some clinical trials conducted in China is weaker than that in trials conducted outside China, leading to the suspicion that this may be caused by differential Glycosylated Hemoglobin (HbA1c) response in the placebo arm of DPP-4 inhibitor clinical trials conducted in China compared to other countries.

**Methods:**

We searched published articles and other documents related to phase III placebo-control trials of DPP-4 inhibitors in Type 2 diabetes mellitus (T2DM). We included studies from different countries and compared those conducted in China to those conducted in other countries. Meta-regression analysis was used to analyze the HbA1c response in the placebo arms.

**Results:**

A total of 66 studies met the inclusion criteria and 10 were conducted within China. There were a total of 8303 participants (mean age 56, male 57 %) in placebo groups. The pooled change in HbA1c for the placebo groups of 10 trials conducted in patients with T2DM in China was 0.26 % (95 % CI [-0.36 %, -0.16 %], *p*-value < 0.001), compared to 0.015 % (95 % CI [-0.05 %, 0.08 %], *p*-value is 0.637) for 56 trials conducted outside of China. The difference of placebo effect between trials conducted in and outside China is -0.273 % (95 % CI [-0.42 %, -0.13 %], *p*-value is less than 0.001) while after excluding trials conducted in Japan, the difference is -0.203 % (95 % CI [-0.35 %, -0.06 %], *p*-value is 0.005). They are both statistically significant.

**Conclusions:**

The meta-analysis in the article demonstrates that there is statistically significant difference in the HbA1c response in the placebo arm of DPP-4 inhibitor clinical trials conducted in China compared to other countries. This differential HbA1c response in the placebo arm should be taken into consideration by both experimenters and medical decision makers when future DPP-4 studies are conducted in China.

## Background

Diabetes has become one of the most threatening non-infectious diseases. The prevention and treatment of diabetes is very important in China because China may have become a country with the largest number of diabetes patients. Most of these patients have type 2 diabetes mellitus (T2DM) [1]. Dipeptidyl peptidase-4 (DPP-4) inhibitors are a class of oral antihyperglycemic agents which has been proved to be better than some traditional treatments in many aspects [2–4]. The *Guideline of Prevention and Treatment for T2DM in China* [5] published by Chinese Diabetes Society places DPP-4 inhibitors second-line with metformin for combination therapy if metformin for monotherapy cannot control glycemic properly. Thus DPP-4 inhibitors are crucial for the treatment of T2DM in China.

However, it has been observed, in some clinical trials conducted in China, the efficacy of DPP-4 inhibitors is weaker than that in trials conducted outside China [6]. It has been suspected that this phenomenon may be caused by a higher placebo effect (i.e., differential HbA1c response in the placebo arm). So far, no one has investigated systematically whether this placebo effect truly exists. If this placebo effect exists, it is problematic for not only the ongoing studies but also further studies. Moreover, this medical information should be considered as an important factor for decision making in conducting DPP-4 trials in China in the future.

Consequently, there is a critical need to investigate the differential HbA1c response in the placebo arm in trials conducted in China. To serve this need, we did a meta-analysis to investigate HbA1c response in the placebo arm in phase III placebo-control clinical trials of DPP-4 inhibitors. We concentrated on the HbA1c response in the placebo arm in trials conducted in China. We also included studies from other countries and compared those conducted in China to those conducted in other countries.

## Methods

### Search strategy and inclusion criteria

We searched EMBASE, PubMed, Google Scholar, ClinicalTrials.gov and PharmaProject for phase III placebo-control clinical trials of DPP-4 inhibitors in T2DM until March 2016. The key words for searching were *sitagliptin*, *saxagliptin*, *vildagliptin*, *linagliptin*, *alogliptin*, *Dipeptidyl peptidase-4 inhibitor* and *DPP-4*. The publications of these trials came from EMBASE, PubMed, and Google. We also included those being completed with results, but unpublished, trials at ClinicalTrials.gov. We browsed the trial list of every drug in PharmaProject to ensure completeness.

The meta-analysis included randomized placebo controlled phase III clinical trials conducted in patients 18 years or older with T2DM on the following DPP-4 inhibitors: Sitagliptin (FDA approved in 2006, CFDA approved in 2009, marketed by Merck & Co. as Januvia), Saxagliptin (FDA approved in 2009, CFDA approved in 2011, marketed by Bristol-Myers Squibb as Onglyza), Vildagliptin (EU approved in 2007, CFDA approved in 2011, marketed by Novartis as Galvus), Linagliptin (FDA approved in 2011, CFDA approved in 2013, marketed by Eli Lilly Co and Boehringer Ingelheim as Trajenta), Alogliptin (FDA approved in 2013, CFDA approved in 2013, marketed by Takeda Pharmaceutical Company as Nesina).

Trials included in this meta-analysis met the following criteria: (1) Only randomized placebo controlled phase III clinical trials are included, (2) DPP-4 inhibitor as monopoly or combination therapy is compared with placebo, (3) patients should be treated for at least 12 weeks, (4) HbA1c is the primary endpoint, (5) the trials were conducted in China with independent results of Chinese patients or conducted outside China without Chinese patients.

### Data extraction and quality evaluation

We extract characteristics and results of trials including the following items: location, experimental drug, combined drug, test duration, the number of patients included, average age, gender, diabetes duration, baseline HbA1c, and the change of HbA1c in control groups.

We used the scoring system developed by Jadad et al. [7] to evaluate the quality of the publications. Randomization, double-blinding, and description of withdraws are considered. Possible scores range from 0 to 5.

### Statistical analysis

The package *Metafor* [8] in R was used to conduct statistical analysis for the HbA1c response in the placebo arm. This package consists of a collection of functions that allow the user to fit fixed-, random-, and mixed-effects models and to carry out meta-regression analysis. Weighted Mean change and 95 % confidence interval for changes from baseline in HbA1c in the placebo control groups were calculated. For studies in China, unless there was heterogeneity, we would use a fixed-effects model. Otherwise a random-effects model would be used. When comparing trials in China with those outside China, a mixed-effects model was performed. We use I^2^ to determine heterogeneity [9] and leave-one-out to perform sensitivity analysis. Publication bias was examined by Egger’s regression test [10].

## Results

### Characteristics of studies

**Fig. 1:**
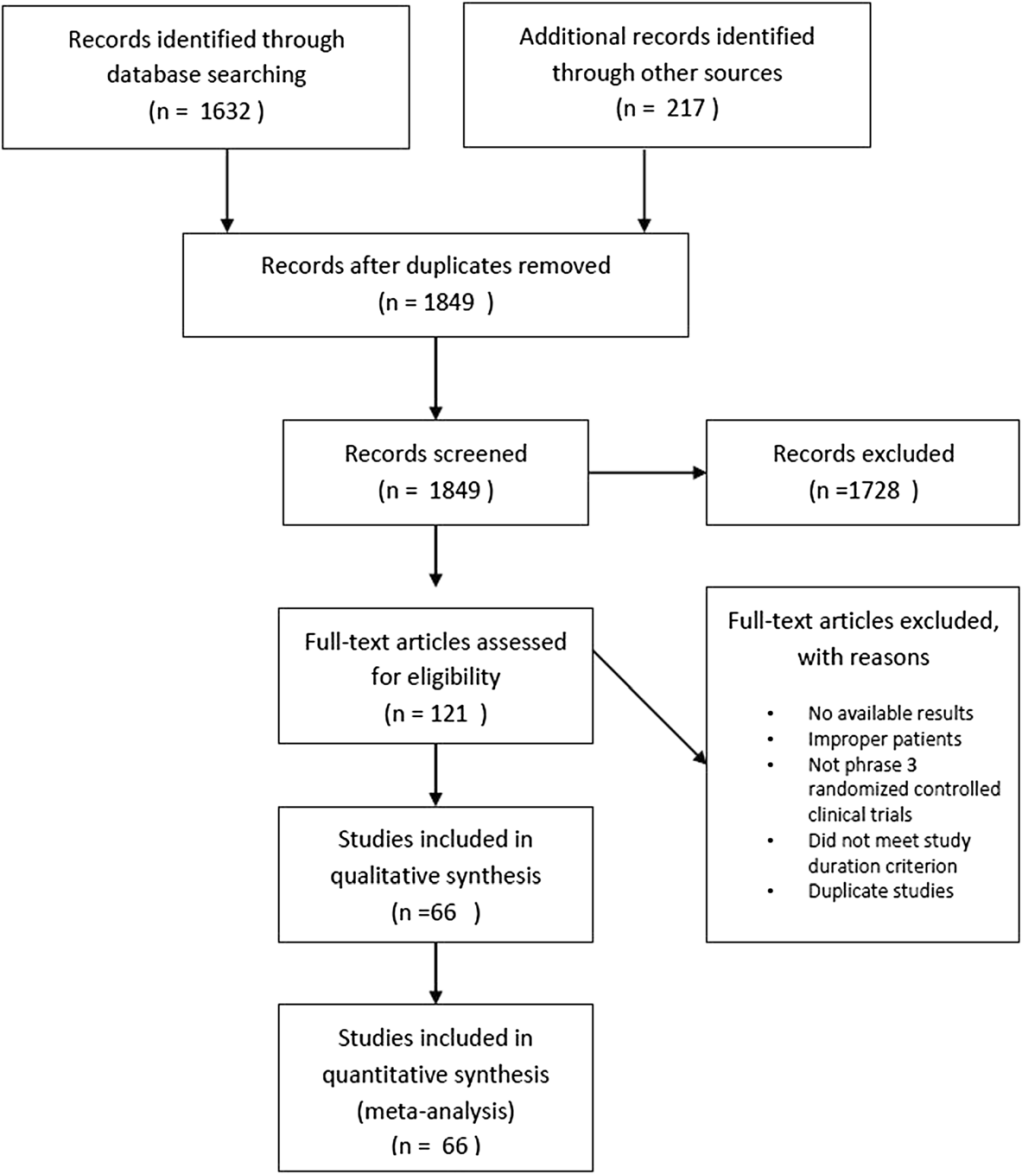
A flow diagram of the selection of eligible studies

A total of 1632 papers and 217 trials were identified. After careful review, 10 studies [6, 11–19] conducted in China were included among which there were 3 unpublished trials [12, 13, 17]. In addition, 56 studies [20–75] conducted outside China were included. Most of these trials including patients coming from different countries, while there were 17 trials [59–75] involving only Japanese patients. There were a total of 8303 participants (mean age 56, male 57 %) in placebo groups. Search results are summarized in Fig. 1.

The summarized information on the included studies is shown in Tables 1 and 2. Table 1 displays the information of studies conducted on Chinese patients in China. There are 4 trials for Sitagliptin, 3 trials for Vildagliptin, 2 trials for Linagliptin and 1trial for Alogliptin. A total of 1634 patients with T2DM in the placebo groups were included. Their average age is between 50 and 60 while the average durations of diabetes are quite different. Average baselines of HbA1c are above 8 %. The treatment time of most studies is 24 weeks while Mohan [14] has 18 weeks and NCT01289119 [17] has 16 weeks. Besides, NCT01076088 [12] and NCT01289119 [17] both have 3 placebo groups.

**Table 1:** Trials conducted in China

Study ID	Location	Drug	Combination therapy	Participants in placebo groups, N	Average age, years	Gender, male, %	Duration of diabetes, years	Baseline HbA1c, %	Change of HbA1c in placebo groups, %	Duration, weeks	Jadad score
Yang [11]	China	Sitagliptin	Metformin	198	55	55	7.30	8.50	-0.14	24	5
NCT01076088 [12]	China	Sitagliptin	None	127	40	68	/	8.97	-0.59	24	/
			Metformin	126	57	55		8.69	-1.29		
			Metformin	124	49	60		8.67	-1.56		
NCT01177384 [13]	China	Sitagliptin	Acarbose	189	57	51	/	8.08	-0.14	24	/
Mohan [14]	China	Sitagliptin	/	82	51	60	1.70	8.60	-0.20	18	5
Pan [6]	China	Vildagliptin	Metformin	144	54	46	5.15	8.01	-0.54	24	3
Yang [15]	China	Vildagliptin	Glimepiride	136	59	58	6.90	8.70	-0.20	24	4
Zeng [16]	China	Linagliptin	Metformin & sulphonylurea	48	57	52	>5	8.13	0.08	24	3
NCT01289119 [17]	China	Alogliptin	None	93	53	58	2.12	/	-0.42	16	/
			Metformin	98	53	49	5.33		-0.22		
			Pioglitazone	63	52	62	4.85		-0.25		
Chen [18]	China	Linagliptin	/	88	54	59	/	8.09	-0.25	24	4
Ning [19]	China	Vildagliptin	Insulin	118	58.5	55	11.4	8.6	-0.22	24	5

**Table 2:** Trials conducted outside China

Study ID	Location	Drug	Combination therapy	Participants in placebo groups, N	Average age, years	Gender, male, %	Duration of diabetes, years	Baseline HbA1c, %	Change of HbA1c in placebo groups, %	Duration, weeks	Jadad score
Rosenstock [20]	non-China	Alogliptin	Insulin	130	55	48	12.2	9.30	-0.13	26	5
Nauck [21]	non-China	Alogliptin	Metformin	104	56	48	6.0	8.00	-0.10	26	5
Raz [22]	non-China	Sitagliptin	/	103	55	63	4.7	8.00	0.12	18	4
Aschner [23]	non-China	Sitagliptin	/	244	54	51	4.6	8.00	0.18	24	4
Hanefeld [24]	non-China	Sitagliptin	/	111	56	63	3.3	7.60	0.12	12	4
Goldstein [25]	non-China	Sitagliptin	Metformin	176	54	53	4.6	8.70	0.17	24	4
Charbonnel [26]	non-China	Sitagliptin	Metformin	237	55	60	6.6	7.98	-0.02	24	4
Raz [27]	non-China	Sitagliptin	Metformin	94	56	42	7.3	9.10	0.00	30	5
Rosenstock [28]	non-China	Sitagliptin	Pioglitazone	178	57	58	6.1	8.02	-0.15	24	4
Hermansen [29]	non-China	Sitagliptin	Glimepiride, Metformin	219	56	53	9.3	8.34	0.28	24	5
Vilsbøll [30]	non-China	Sitagliptin	Insulin	319	57	53	12.0	8.60	0.00	24	5
Scott [31]	non-China	Sitagliptin	/	125	55	62	4.8	7.90	0.23	12	5
Scott [32]	non-China	Sitagliptin	Metformin	92	55	59	5.4	7.70	-0.22	18	4
Ristic [33]	non-China	Vildagliptin	/	58	55	57	2.3	7.76	-0.13	12	3
Dejager [34]	non-China	Vildagliptin	/	94	52	48	1.6	8.40	-0.30	24	4
Pi-Sunyer [35]	non-China	Vildagliptin	/	92	52	54	2.5	8.50	0.00	24	4
Bosi [36]	non-China	Vildagliptin	Metformin	130	55	53	6.2	8.30	0.20	24	3
Garber [37]	non-China	Vildagliptin	Pioglitazone	138	55	51	4.8	8.70	-0.30	24	4
Garber [38]	non-China	Vildagliptin	Glimepiride	144	58	58	7.8	8.50	0.07	24	5
Fonseca [39]	non-China	Vildagliptin	Insulin	152	59	55	14.9	8.40	-0.20	24	4
Defronzo [40]	non-China	Saxagliptin	Metformin	179	55	54	6.7	8.10	0.13	24	4
Del Prato [41]	non-China	Linagliptin	/	167	54	47	/	8.00	0.25	24	4
Taskinen [42]	non-China	Linagliptin	Metformin	177	57	57	/	8.02	0.15	24	4
Moses [43]	non-China	Sitagliptin	Sulfonylurea, Metformin	212	55.4	46	8	8.4	-0.16	24	5
Laakso [44]	non-China	Linagliptin	Glimepiride	120	66.6	63.4	/	8.1	-0.11	12	3
White [45]	non-China	Saxagliptin	Metformin	84	56.6	52.3	6.2	7.97	-0.22	12	5
Moses [46]	non-China	Saxagliptin	Sulfonylurea, Metformin	127	56.8	57.8	/	8.2	-0.08	24	4
Bajaj [47]	non-China	Linagliptin	Metformin, Pioglitazone	89	55.2	55.1	/	8.47	-0.27	24	4
Fonseca [48]	non-China	Sitagliptin	Metformin, Pioglitazone	153	56.4	62.8	10.2	8.6	-0.4	26	5
Kothny [49]	non-China	Vildagliptin	Insulin	221	59.1	52	13.2	8.8	-0.1	24	4
Dobs [50]	non-China	Sitagliptin	Metformin, Osiglitazone	88	54.8	60	9.4	8.7	-0.3	18	5
Lewin [51]	non-China	Linagliptin	Sulfonylurea	82	56.2	61.9	/	8.6	-0.07	18	4
Barnett [52]	non-China	Linagliptin	/	73	56.7	43.4	/	8.1	0.21	18	5
Forst [53]	non-China	Linagliptin	Metformin	70	60.1	62	6.2	8.4	0.24	12	4
Nowicki [54]	non-China	Saxagliptin	/	83	66.2	48.2	18.2	8.09	-0.44	12	5
Gomis [55]	non-China	Linagliptin	Pioglitazone	128	57.1	65.4	/	8.58	-0.56	24	4
Hollander [56]	non-China	Saxagliptin	Thiazolidinedione	180	54.1	46.2	5.1	8.2	-0.3	24	4
Pratley [57]	non-China	Alogliptin	Glyburide	99	57.1	51.5	7.7	8	0.01	26	4
Pratley [58]	non-China	Vildagliptin	/	26	52.8	50	3.5	8.1	0	12	5
Nonaka [59]	Japan	Sitagliptin	/	76	55	66	4.1	7.70	0.41	12	5
Kikuchi [60]	Japan	Vildagliptin	/	20	62	55	7.2	7.30	0.28	12	4
Iwamoto [61]	Japan	Sitagliptin	/	73	60	69	6.4	7.74	0.28	12	4
Kikuchi [62]	Japan	Vildagliptin	Glimepiride	100	60	69	9.8	8.00	-0.06	12	4
Kaku [63]	Japan	Alogliptin	Pioglitazone	115	60	66	6.7	7.92	-0.19	12	4
Kashiwagi [64]	Japan	Sitagliptin	Pioglitazone	68	59	72	7.6	8.00	0.40	12	5
Seino [65]	Japan	Alogliptin	Voglibose	75	62	64	7.5	8.12	0.04	12	5
Kawamori [66]	Japan	Linagliptin	/	80	60	71	5.0	7.95	0.63	12	4
Seino [67]	Japan	Alogliptin	Metformin	100	52	72	6.0	8.00	0.21	12	5
Seino [68]	Japan	Alogliptin	Sulfonylurea	103	60	69	9.4	8.62	0.35	12	4
Kadowaki [69]	Japan	Sitagliptin	Metformin	72	57	68	7.3	8.40	0.30	12	5
Kaku [70]	Japan	Alogliptin	Insulin	89	62	53	14.5	8.43	-0.31	12	4
Odawara [71]	Japan	Vildagliptin	Metformin	70	58	69	7.0	8.00	-0.10	12	4
Hirose [72]	Japan	Vildagliptin	Insulin	75	60.1	71.2	12.9	8.1	-0.11	12	5
Tajima [73]	Japan	Sitagliptin	Voglibose	63	58.6	71.5	/	7.9	0.2	12	4
Kadowaki [74]	Japan	Sitagliptin	Insulin	128	60.2	58.4	14	8.9	0.3	16	4
Tajima [75]	Japan	Sitagliptin	Glimepiride	64	61	58.2	7.9	8.3	0.3	12	5

Table 2 shows the 56 trials conducted on non-Chinese patients outside China. There are 21 trials for Sitagliptin, 13 trials for Vildagliptin, 9 trials for Linagliptin, 8 trials for Alogliptin and 5 trials for Saxagliptin. A total of 6669 patients with T2DM in the placebo groups were included. There is no significant difference in average age between patients in Tables 1 and 2, respectively, whereas the Japanese patients seem a little older than others. As for the duration of diabetes, patients in studies using insulin as the combination therapy suffered longer than others. The baselines of HbA1c range from 7.30 % to 9.30 %, and the variation is greater than those in Table 1. All the trials conducted in Japan treated patients for 12 weeks except Kadowaki 2013, and the treatment duration of most other studies in Table 2 is 24 weeks.

### Differential HbA1c response in the placebo arm

We analyzed the placebo effect in all the trials, focusing on HbA1c change from baseline in controlled groups. Generally, placebo should not have a significant effect on HbA1c, even if there was combination therapy. The general HbA1c change from baseline in placebo controlled groups should be close to 0. However, it has been observed that there is a high placebo effect in some trials conducted in China [6]. We focused on HbA1c change from baseline in placebo controlled groups in trials conducted in China. We first summarized the HbA1c response in the placebo arm in trials conducted in China and then made a comparison to those conducted outside China.

**Fig. 2:**
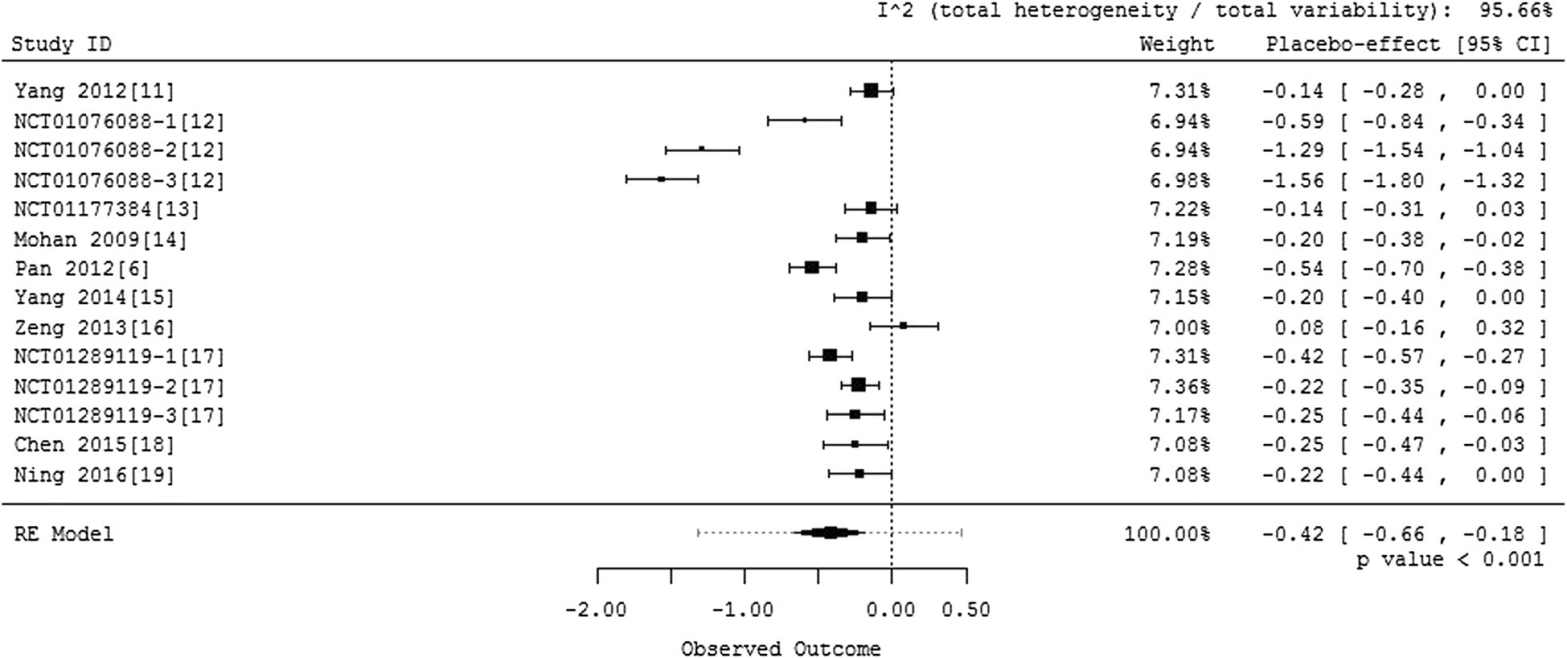
Forest plot for all placebo groups in trials conducted in China

Among 10 randomized placebo-controlled phase III clinical trials of DPP-4 inhibitors conducted in patients with T2DM in China, NCT01076088 [12] and NCT01289119 [17] have 3 placebo controlled groups with different combination therapy respectively. Thus totally 14 groups were included in the meta-analysis. Since there was substantial heterogeneity, random-effects model was performed. The weighted mean change from baseline was calculated and forest plot was drawn. Results are shown in Fig. 2. The results show that HbA1c is declined by 0.42 % with a *p*-value less than 0.001 in the placebo arm of randomized placebo controlled phase III clinical trials of DPP-4 inhibitors conducted in patients with T2DM in China. The 95 % confidence interval is (-0.66 %, -0.18 %).

**Fig. 3:**
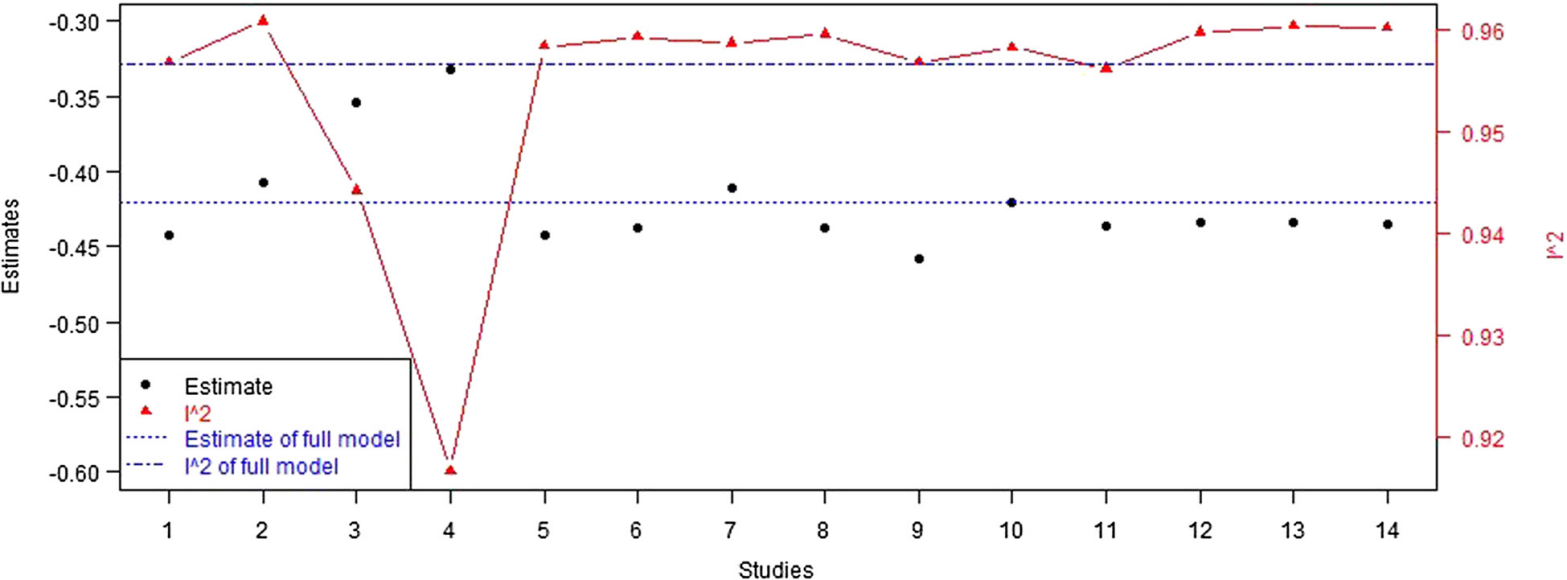
Results of leave-one-out sensitivity analysis

We noted that the heterogeneity index I^2^ of this model is significantly large. Thus we performed a leave-one-out sensitivity analysis to detect the influence of each study. Each time we left one group out, then fitted the same model. We got the summary estimates and I^2^ of 14 models with results shown in Fig. 3. We found that the estimates of models that left out NCT01076088-2 [12] or NCT01076088-3 [12] are significant different from those in the full model that included all the 14 groups. The other groups show less heterogeneity. In addition, from Fig. 2, we see that the placebo effects of NCT01076088-2 [12] and NCT01076088-3 [12] are much higher than others, both larger than 1 %. In these two groups, metformin was used as combination therapy, patients’ baseline HbA1c were higher than average, and there were no information about the duration of diabetes. These might cause the significant difference in HbA1c decline between the two studies and others.

**Fig. 4:**
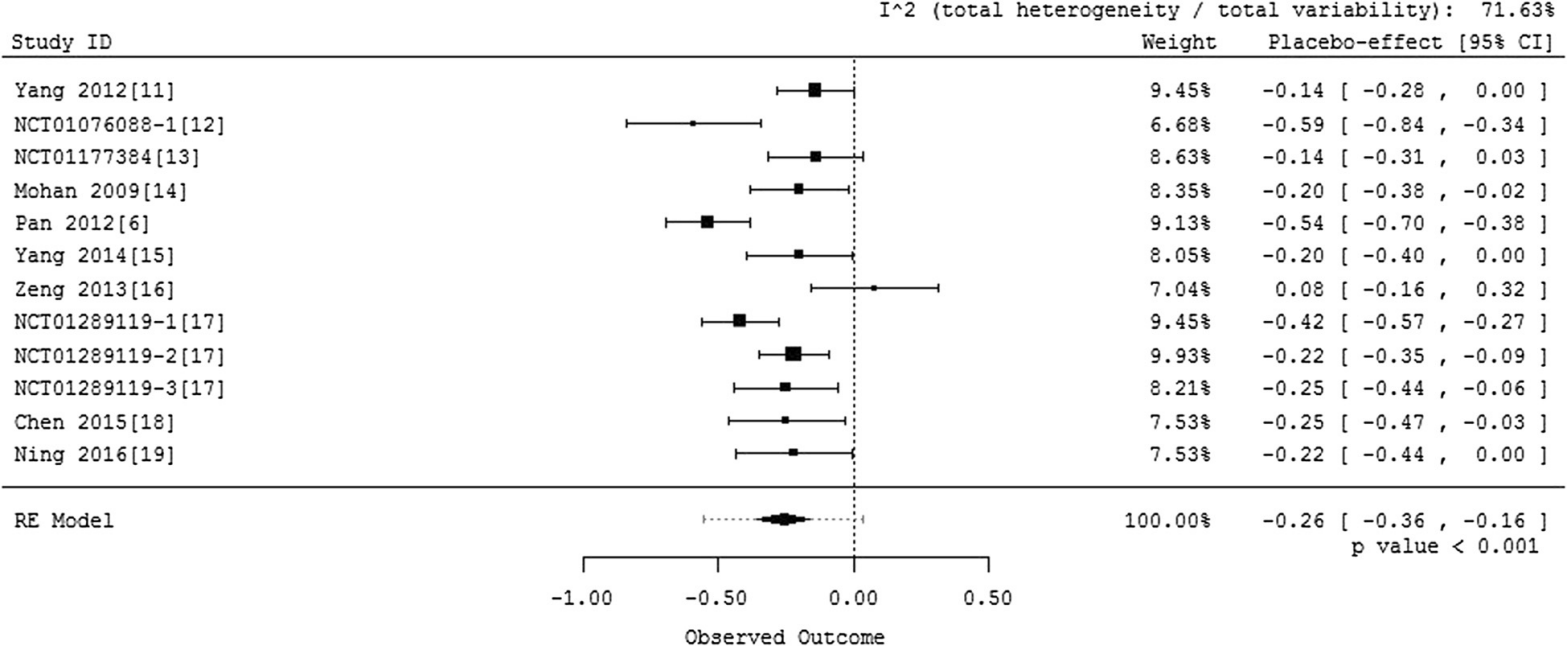
Forest plot for placebo groups in trials conducted in China after excluding two extreme groups

To decrease the heterogeneity, we further performed a random-effect model excluding these two groups. The weighted mean change from baseline was calculated and a forest plot was drawn. Results are shown in Fig. 4. In this model, I^2^ declines to 71.63 %. We also performed a leave-one-out sensitivity analysis on this model. While leaving out some studies would decrease I^2^, the estimates wouldn’t change much. The results based on the model excluding NCT01076088-2 [12] and NCT01076088-3 [12] indicate that, HbA1c in placebo controlled groups declined by 0.26 % with 95 % confidence interval being (-0.36 %, -0.16 %) and *p*-value less than 0.001 (Fig. 4). Egger’s regression test shows there is no publication bias (*p*-value is 0.802).

The results based on either of the two models (i.e., the full model and the model excluding two heterogeneous studies) show a significant placebo effect in the placebo arm in the same direction as the DDP-4 inhibitor’s effect. These results provide statistically significant evidence that the placebo effect is not 0 in randomized placebo controlled phase III clinical trials of DPP-4 inhibitors conducted in patients with T2DM in China.

Considering minimizing the impact of heterogeneity in the analysis, the analysis based on the model excluding the two heterogeneous studies may be more reliable. Thus, for future reference, we may conclude that placebo decreases HbA1c by 0.26 % (95 % CI [-0.36 %, -0.16 %] and *p*-value less than 0.001) generally in trials conducted in Chinese patients in China (Fig. 4).

We compared trials conducted in China with those outside China using a mixed-effects model. Table 3 shows the analysis results. The model is fitted with location as a moderator. We treat location as a binary variable, 1 for China and 0 for non-China.

**Table 3:** Mixed-effects model 1 (k = 68)

		Standard error	z value	*p* value	Lower 95 % confidence interval	Upper 95 % confidence interval
Intercept	0.015	0.031	0.472	0.637	-0.047	0.076
Factor(location)1	-0.273	0.076	-3.612	<.001	-0.421	-0.125

The model is fitted with location as moderator. Location was regarded as binary variable, 1 for China and 0 for non-China

The intercept, 0.015 % (95 % CI [-0.05 %, 0.08 %], *p*-value is 0.637), shows that the HbA1c response in the placebo arm of trials conducted outside China (location = 0) is close to 0. The coefficient for location, -0.273 % (95 % CI [-0.42 %, -0.13 %], *p*-value is less than 0.001), shows a large difference of HbA1c response in the placebo arm between trials conducted in China (location = 1) and those outside China. The difference is statistically significant.

In addition, as trials conducted in Japan have shorter test duration and in some trials placebo significantly increased HbA1c, we performed another model treating Japan as a separate group. Table 4 shows the analysis results. The model is fitted with location as a moderator, 1 for China, 2 for Japan and 0 for others.

**Table 4:** Mixed-effects model 2 (k = 68)

		Standard error	z value	*p* value	Lower 95 % confidence interval	Upper 95 % confidence interval
Intercept	-0.055	0.035	-1.565	0.118	-0.123	0.014
Factor(location)1	-0.203	0.073	-2.803	0.005	-0.346	-0.061
Factor(location)2	0.22	0.062	3.546	<.001	0.098	0.342

The model is fitted with location as moderator. Location was regarded as categorical variable, 1 for China, 2 for Japan and 0 for others

This model shows that the HbA1c response in the placebo arm in trials conducted in countries except China and Japan was close to 0 (-0.055 with *p*-value 0.118). Trials conducted in Japan had a reverse placebo effect which means placebo increased HbA1c by 0.22 % (95 % CI [0.10 %, 0.34 %], *p*-value is less than 0.001). Finally, the model shows the difference of HbA1c response in the placebo arm between trials conducted in China and other countries except Japan is -0.203 % (95 % CI [-0.35 %, -0.06 %]) with a *p*-value of 0.005. The difference is also statistically significant.

## Discussion

DPP-4 inhibitors are an important class of oral antihyperglycemic agents [2–4]. Until now five DPP-4 inhibitors, sitagliptin, saxagliptin, vildagliptin, linagliptin, alogliptin, have been approved for marketing by CFDA. A large number of trials have been conducted in China determining the efficacy of these drugs in Chinese patients. For example, in a 24-week, randomized, double-blind, placebo-controlled study with 438 Chinese T2DM patients, Pan [6] discovered that the adjusted mean change in HbA1c at endpoint was −1.05 ± 0.08 %, −0.92 ± 0.08 % and −0.54 ± 0.08 % in patients receiving vildagliptin 50 mg bid, 50 mg qd and placebo, respectively. In this study, the 95 % confidence interval for the HbA1c response in the placebo arm is (-0.70, -0.38), indicating an HbA1 decline in the placebo arm. Similar HbA1 decline was discovered in a fair number of other trials in Chinese patients such as NCT01076088 [12] and NCT01289119 [17]. There are a few trials with the 95 % confidence interval of HbA1 in the placebo arm covering 0 such as (-0.28, 0) in Yang [11] and (-0.16, 0.32) in Zeng [16]. However, no trial has shown a significant HbA1c increase in the placebo arm in trials with Chinese patients. Therefore, there is a suspicion that there is an HbA1c decline in the placebo arm of DPP-4 inhibitor clinical trials conducted in China. Is that suspicion true? No one has addressed this question systematically yet. Therefore, in this article, we use a systematic meta-analysis approach to address this question.

The meta-analysis shows that, HbA1c in the placebo arm declined by 0.26 % (95 % CI [-0.36 %, -0.16 %] and *p*-value less than 0.001) in trials of DPP-4 inhibitors conducted in patients with T2DM in China, whereas the placebo effect of those conducted outside China is close to 0. The difference of HbA1c in the placebo arm between trials conducted in China and outside China is -0.273 % (95 % CI [-0.42 %, -0.13 %], *p*-value is less than 0.001). After excluding trials conducted in Japan, the difference is -0.203 % (95 % CI [-0.35 %, -0.06 %]) with a *p*-value of 0.005. They are both statistically significant. Therefore, after we investigated the placebo effect of randomized placebo controlled phase III clinical trials of DPP-4 inhibitors conducted in patients with T2DM in China, we concluded that there was statistically significant difference in response in the placebo arm between trials conducted in China and outside China. This difference of HbA1c decline in the placebo arm should be taken into account in future studies in China.

There may be various reasons for this high placebo effect. However, what these reasons exactly are is unknown. Although the investigation of these reasons is not the purpose in this article, we have the following two major guesses. First, the practical process of these trials could cause a bias. Most of the trials provided the participants the significant benefit to obtain more resource of medical care, esp. in China. Because of no established PCP system, low awareness of diabetes management, China has most diabetes patients in the world [5] but much lower health workers/patient ratio than Europe, USA and Japan [76]. Clinical trials could have obvious impact on management of diabetes, and thus cause better blood glucose control even in placebo arm. Second, Traditional Chinese Medicine (TCM) could play role. There is evidence that the use of some TCM herbs can reduce hyperglycemia [77]. Among these herbs many are used commonly in diet or drinks. Normally in study protocol, herbs in diet were not clearly inhibited. Other reasons may include life style and culture that are unique in China.

The results on the placebo effect on other countries may also provide values for future trials and medical interpretation. For example, clinical trials conducted in Japan had a statistically significant reverse placebo effect, which may be important information for conducting future DDP-4 trials as well as for interpreting trial results in Japan.

## Conclusions

The meta-analysis in the article demonstrates that there are significant differences in response in the placebo group of DPP-4 trials conducted in China compared to those conducted outside of China. This difference may give some clue to why the efficacy of these drugs is less statistically significant in trials conducted in China. More importantly, this difference in response in the placebo group should be taken into account for future DDP-4 trials conducted in China. In addition, the difference in placebo should be carefully considered by medical decision makers when future DPP-4 studies are conducted in China.

## Abbreviations

CFDA: China food and drug administration
DPP-4: Dipeptidyl peptidase-4
EU: European union
FDA: Food and drug administration
HbA1c: Glycosylated hemoglobin
T2DM: Type 2 diabetes mellitus

## References

[1] Guariguata L, Whiting DR, Hambleton I, Beagley J, Linnenkamp U, Shaw JE (2014). Global estimates of diabetes prevalence for 2013 and projections for 2035. Diabetes Res Clin Pract.

[2] Campbell RK (2006). Rationale for Dipeptidyl Peptidase 4 Inhibitors: A New Class of Oral Agents for the Treatment of Type 2 Diabetes Mellitus. Ann Pharmacother.

[3] Yousefzadeh P, Yousefzadeh P, Wang X, Wang X (2013). The Effects of Dipeptidyl Peptidase-4 Inhibitors on Cardiovascular Disease Risks in Type 2 Diabetes Mellitus. J Diabetes Res.

[4] Rosenstock J, Zinman B (2007). Dipeptidyl peptidase-4 inhibitors and the management of type 2 diabetes mellitus. Curr Opin Endocrinol Diabetes Obes.

[5] China Diabetes Society (2014). Guideline of Prevention and Treatment for T2DM in China (2013). Chin J Diabetes Mellitus.

[6] Pan C, Xing X, Han P, Zheng S, Ma J, Liu J, Lv X, Lu J, Bader G (2012). Efficacy and tolerability of vildagliptin as add‐on therapy to metformin in Chinese patients with type 2 diabetes mellitus. Diabetes Obes Metab.

[7] Jadad AR, Moore RA, Carroll D, Jenkinson C, Reynolds DJM, Gavaghan DJ, McQuay HJ (1996). Assessing the quality of reports of randomized clinical trials: Is blinding necessary?. Control Clin Trials.

[8] Viechtbauer W. metafor: Meta-Analysis Package for R. http://www.metafor-project.org/doku.php. Accessed Sept 2014.

[9] Higgins JPT, Thompson SG (2002). Quantifying heterogeneity in a meta-analysis. Statist Med.

[10] Egger M, Smith GD, Schneider M, Minder C (1997). Bias in meta-analysis detected by a simple, graphical test. BMJ.

[11] Yang W, Guan Y, Shentu Y, Li Z, Johnson Levonas AO, Engel SS, Kaufman KD, Goldstein BJ, Alba M (2012). The addition of sitagliptin to ongoing metformin therapy significantly improves glycemic control in Chinese patients with type 2 diabetes*†. J Diabetes.

[12] Safety and Efficacy of Co-Administration of Sitagliptin and Metformin in China (MK-0431-121). https://clinicaltrials.gov/ct2/show/NCT01076088. Accessed Aug 2014.

[13] Clinical Trial to Evaluate the Safety and Efficacy of the Addition of Sitagliptin in Participants With Type 2 Diabetes Mellitus Receiving Acarbose Monotherapy (MK-0431-130). http://clinicaltrials.gov/ct2/show/NCT01177384. Accessed Aug 2014.

[14] Mohan V, Yang W, Son H-Y, Xu L, Noble L, Langdon RB, Amatruda JM, Stein PP, Kaufman KD (2009). Efficacy and safety of sitagliptin in the treatment of patients with type 2 diabetes in China, India, and Korea. Diabetes Res Clin Pract.

[15] Yang W, Xing X, Lv X, Li Y, Ma J, Yuan G, Sun F, Wang W, Woloschak M, Lukashevich V, Kozlovski P, Kothny W (2015). Vildagliptin added to sulfonylurea improves glycemic control without hypoglycemia and weight gain in Chinese patients with type 2 diabetes mellitus. J Diabetes.

[16] Zeng Z, Yang J-K, Tong N, Yan S, Zhang X, Gong Y, Woerle H-J (2013). Efficacy and safety of linagliptin added to metformin and sulphonylurea in Chinese patients with type 2 diabetes: a sub-analysis of data from a randomised clinical trial. Curr Med Res Opin.

[17] Efficacy and Safety of Alogliptin in Participants With Type 2 Diabetes. http://clinicaltrials.gov/ct2/show/NCT01289119. Accessed Aug 2014.

[18] Chen Y, Ning G, Wang C, Gong Y, Patel S, Zhang C, Izumoto T, Woerle H-J, Wang W (2015). Efficacy and safety of linagliptin monotherapy in Asian patients with inadequately controlled type 2 diabetes mellitus: A multinational, 24-week, randomized, clinical trial. J Diabetes Investig.

[19] Ning G, Wang W, Li L, Ma J, Lv X, Yang M, Wang W, Woloschak M, Lukashevich V, Kothny W (2016). Vildagliptin as add-on therapy to insulin improves glycemic control without increasing risk of hypoglycemia in Asian, predominantly Chinese, patients with type 2 diabetes mellitus. J Diabetes.

[20] Rosenstock J, Rendell MS, Gross JL, Fleck PR, Wilson CA, Mekki Q (2009). Alogliptin added to insulin therapy in patients with type 2 diabetes reduces HbA1c without causing weight gain or increased hypoglycaemia. Diabetes Obes Metab.

[21] Nauck MA, Ellis GC, Fleck PR, Wilson CA, Mekki Q (2009). Efficacy and safety of adding the dipeptidyl peptidase-4 inhibitor alogliptin to metformin therapy in patients with type 2 diabetes inadequately controlled with metformin monotherapy: a multicentre, randomised, double-blind, placebo-controlled study. Int J Clin Pract.

[22] Raz I, Hanefeld M, Xu L, Caria C, Williams-Herman D, Khatami H, Group SS0 (2006). Efficacy and safety of the dipeptidyl peptidase-4 inhibitor sitagliptin as monotherapy in patients with type 2 diabetes mellitus. Diabetologia.

[23] Aschner P, Kipnes MS, Lunceford JK (2006). Effect of the dipeptidyl peptidase-4 inhibitor sitagliptin as monotherapy on glycemic control in patients with type 2 diabetes. Diabetes Care.

[24] Hanefeld M, Herman GA, Wu M, Mickel C, Sanchez M, Stein PP, investigators OBOTSS0 (2007). Once-daily sitagliptin, a dipeptidyl peptidase-4 inhibitor, for the treatment of patients with type 2 diabetes. Curr Med Res Opin.

[25] Goldstein BJ, Feinglos MN, Lunceford JK (2007). Effect of initial combination therapy with sitagliptin, a dipeptidyl peptidase-4 inhibitor, and metformin on glycemic control in patients with type 2 diabetes. Diabetes Care.

[26] Charbonnel B, Karasik A, Liu J, Wu M, Meininger G (2006). Efficacy and Safety of the Dipeptidyl Peptidase-4 Inhibitor Sitagliptin Added to Ongoing Metformin Therapy in Patients With Type 2 Diabetes Inadequately Controlled With Metformin Alone. Dia Care.

[27] Raz I, Chen Y, Wu M, Hussain S, Kaufman KD, Amatruda JM, Langdon RB, Stein PP, ALBA M (2008). Efficacy and safety of sitagliptin added to ongoing metformin therapy in patients with type 2 diabetes. Curr Med Res Opin.

[28] Rosenstock J, Brazg R, Andryuk PJ, Lu K, Stein P (2006). Efficacy and safety of the dipeptidyl peptidase-4 inhibitor sitagliptin added to ongoing pioglitazone therapy in patients with type 2 diabetes: A 24-week, multicenter, randomized, double-blind, placebo-controlled, parallel-group study. Clin Ther.

[29] Hermansen K, Kipnes M, Luo E, Fanurik D, Khatami H, Stein P (2007). Efficacy and safety of the dipeptidyl peptidase-4 inhibitor, sitagliptin, in patients with type 2 diabetes mellitus inadequately controlled on glimepiride alone or on glimepiride and metformin. Diabetes Obes Metab.

[30] Vilsbøll T, Rosenstock J, Yki Järvinen H, Cefalu WT, Chen Y, Luo E, Musser B, Andryuk PJ, Ling Y, Kaufman KD, Amatruda JM, Engel SS, Katz L (2010). Efficacy and safety of sitagliptin when added to insulin therapy in patients with type 2 diabetes. Diabetes Obes Metab.

[31] Scott R, Wu M, Sanchez M, Stein P (2007). Efficacy and tolerability of the dipeptidyl peptidase-4 inhibitor sitagliptin as monotherapy over 12 weeks in patients with type 2 diabetes. Int J Clin Pract.

[32] Scott R, Loeys T, Davies MJ, Engel SS (2008). Efficacy and safety of sitagliptin when added to ongoing metformin therapy in patients with type 2 diabetes*. Diabetes Obes Metab.

[33] Ristic S, Byiers S, Foley J, Holmes D (2005). Improved glycaemic control with dipeptidyl peptidase-4 inhibition in patients with type 2 diabetes: vildagliptin (LAF237) dose response. Diabetes Obes Metab.

[34] Dejager S, Razac S, Foley JE, Schweizer A. Vildagliptin in drug-na ï ve patients with type 2 diabetes: a 24-week, double-blind, randomized, placebo-controlled, multiple-dose study. Horm Metab Res. 2007. 10.1055/s-2007-970422 17373638

[35] Pi-Sunyer FX, Schweizer A, Mills D, Dejager S (2007). Efficacy and tolerability of vildagliptin monotherapy in drug-naïve patients with type 2 diabetes. Diabetes Res Clin Pract.

[36] Bosi E, Camisasca RP, Collober C, Rochotte E, Garber AJ (2007). Effects of Vildagliptin on Glucose Control Over 24 Weeks in Patients With Type 2 Diabetes Inadequately Controlled With Metformin. Dia Care.

[37] Garber AJ, Schweizer A, Baron MA, Rochotte E, Dejager S (2007). Vildagliptin in combination with pioglitazone improves glycaemic control in patients with type 2 diabetes failing thiazolidinedione monotherapy: a randomized, placebo-controlled study*. Diabetes Obes Metab.

[38] Garber AJ, Foley JE, Banerji MA, Ebeling P, Gudbjörnsdottir S, Camisasca RP, Couturier A, Baron MA (2008). Effects of vildagliptin on glucose control in patients with type 2 diabetes inadequately controlled with a sulphonylurea*. Diabetes Obes Metab.

[39] Fonseca V, Schweizer A, Albrecht D, Baron MA, Chang I, Dejager S (2007). Addition of vildagliptin to insulin improves glycaemic control in type 2 diabetes. Diabetologia.

[40] DeFronzo RA, Hissa MN, Garber AJ, Gross JL, Duan RY, Ravichandran S, Chen RS, Group FTS0S (2009). The Efficacy and Safety of Saxagliptin When Added to Metformin Therapy in Patients With Inadequately Controlled Type 2 Diabetes With Metformin Alone. Dia Care.

[41] Del Prato S, Barnett AH, Huisman H, Neubacher D, Woerle HJ, Dugi KA (2011). Effect of linagliptin monotherapy on glycaemic control and markers of β-cell function in patients with inadequately controlled type 2 diabetes: a randomized controlled trial. Diabetes Obes Metab.

[42] Taskinen MR, Rosenstock J, Tamminen I, Kubiak R, Patel S, Dugi KA, Woerle HJ (2011). Safety and efficacy of linagliptin as add-on therapy to metformin in patients with type 2 diabetes: a randomized, double-blind, placebo-controlled study. Diabetes Obes Metab.

[43] Moses RG, Round E, Shentu Y, Golm GT, O’neill EA, Gantz I, Engel SS, Kaufman KD, Goldstein BJ. A randomized clinical trial evaluating the safety and efficacy of sitagliptin added to the combination of sulfonylurea and metformin in patients with type 2 diabetes mellitus and inadequate glycemic control. J Diabetes. 2015. doi:10.1111/1753-0407.12351. 10.1111/1753-0407.12351 26625270

[44] Laakso M, Rosenstock J, Groop PH, Barnett AH (2015). Treatment with the dipeptidyl peptidase-4 inhibitor linagliptin or placebo followed by glimepiride in patients with type 2 diabetes with moderate to severe renal impairment: a 52-week, randomized, double-blind clinical trial. Diabetes Care.

[45] White JL, Buchanan P, Li J, Frederich R (2014). A randomized controlled trial of the efficacy and safety of twice-daily saxagliptin plus metformin combination therapy in patients with type 2 diabetes and inadequate glycemic control on metformin monotherapy. BMC Endocr Disord.

[46] Moses RG, Kalra S, Brook D, Sockler J, Monyak J, Visvanathan J, Montanaro M, Fisher SA (2014). A randomized controlled trial of the efficacy and safety of saxagliptin as add-on therapy in patients with type 2 diabetes and inadequate glycaemic control on metformin plus a sulphonylurea. Diabetes Obes Metab.

[47] Bajaj M, Gilman R, Patel S, Kempthorne Rawson J, Lewis D’Agostino D, Woerle HJ (2014). Linagliptin improved glycaemic control without weight gain or hypoglycaemia in patients with Type 2 diabetes inadequately controlled by a combination of metformin and pioglitazone: a 24-week randomized, double-blind study. Diabet Med.

[48] Fonseca V, Staels B, Morgan JD, Shentu Y, Golm GT, Johnson Levonas AO, kaufman KD, Goldstein BJ, Steinberg H (2013). Efficacy and safety of sitagliptin added to ongoing metformin and pioglitazone combination therapy in a randomized, placebo-controlled, 26-week trial in patients with type 2 diabetes. J Diabetes Complicat.

[49] Kothny W, Foley J, Kozlovski P, Shao Q, Gallwitz B, Lukashevich V (2013). Improved glycaemic control with vildagliptin added to insulin, with or without metformin, in patients with type 2 diabetes mellitus. Diabetes Obes Metab.

[50] Dobs AS, Goldstein BJ, Aschner P, Horton ES, Umpierrez GE, Duran L, Hill JS, Chen Y, Golm GT, Langdon RB, Williams Herman DE, Kaufman KD, Amatruda JM, Ferreira JCA (2013). Efficacy and safety of sitagliptin added to ongoing metformin and rosiglitazone combination therapy in a randomized placebo-controlled 54-week trial in patients with type 2 diabetes. J Diabetes.

[51] Lewin AJ, Arvay L, Liu D, Patel S, Eynatten Von M, Woerle H-J (2012). Efficacy and Tolerability of Linagliptin Added to a Sulfonylurea Regimen in Patients With Inadequately Controlled Type 2 Diabetes Mellitus: An 18-Week, Multicenter, Randomized, Double-Blind, Placebo-Controlled Trial. Clin Ther.

[52] Barnett AH, Patel S, Harper R, Toorawa R, Thiemann S, Eynatten M, Woerle HJ (2012). Linagliptin monotherapy in type 2 diabetes patients for whom metformin is inappropriate: an 18-week randomized, double-blind, placebo-controlled phase III trial with a 34-week active-controlled extension. Diabetes Obes Metab.

[53] Forst T, Uhlig Laske B, Ring A, Graefe Mody U, Friedrich C, Herbach K, Woerle HJ, Dugi KA (2010). Linagliptin (BI 1356), a potent and selective DPP-4 inhibitor, is safe and efficacious in combination with metformin in patients with inadequately controlled Type 2 diabetes. Diabet Med.

[54] Nowicki M, Rychlik I, Haller H, Warren ML, Suchower L, Gause Nilsson I (2011). Saxagliptin improves glycaemic control and is well tolerated in patients with type 2 diabetes mellitus and renal impairment. Diabetes Obes Metab.

[55] Gomis R, Espadero RM, Jones R, Woerle HJ, Dugi KA (2011). Efficacy and safety of initial combination therapy with linagliptin and pioglitazone in patients with inadequately controlled type 2 diabetes: a randomized, double-blind, placebo-controlled study. Diabetes Obes Metab.

[56] Hollander P, Li J, Allen E, Chen R (2011). Saxagliptin Added to a Thiazolidinedione Improves Glycemic Control in Patients with Type 2 Diabetes and Inadequate Control on Thiazolidinedione Alone. J Clin Endocrinol Metab.

[57] Pratley RE, Kipnes MS, Fleck PR, Wilson C, Mekki Q (2009). Efficacy and safety of the dipeptidyl peptidase-4 inhibitor alogliptin in patients with type 2 diabetes inadequately controlled by glyburide monotherapy. Diabetes Obes Metab.

[58] Pratley RE, Holmes D. Twelve − week Monotherapy with the DPP− 4 Inhibitor Vildagliptin Improves Glycemic Control in Subjects with Type 2 Diabetes. Horm Metab Res. 2006. 10.1055/s-2006-944546 16823726

[59] Nonaka K, Kakikawa T, Sato A, Okuyama K, Fujimoto G, Kato N, Suzuki H, Hirayama Y, Ahmed T, Davies MJ, Stein PP (2008). Efficacy and safety of sitagliptin monotherapy in Japanese patients with type 2 diabetes. Diabetes Res Clin Pract.

[60] Kikuchi M, Abe N, Kato M, Terao S, Mimori N, Tachibana H (2009). Vildagliptin dose-dependently improves glycemic control in Japanese patients with type 2 diabetes mellitus. Diabetes Res Clin Pract.

[61] Iwamoto Y, Taniguchi T, Nonaka K, Okamoto T, Okuyama K, Ferreira JCA, Amatruda J (2010). Dose-ranging efficacy of sitagliptin, a dipeptidyl peptidase-4 inhibitor, in Japanese patients with type 2 diabetes mellitus. Endocr J.

[62] Kikuchi M, Haneda M, Koya D, Tobe K, Onishi Y, Couturier A, Mimori N, Inaba Y, Goodman M (2010). Efficacy and tolerability of vildagliptin as an add-on to glimepiride in Japanese patients with Type 2 diabetes mellitus. Diabetes Res Clin Pract.

[63] Kaku K, Itayasu T, Hiroi S, Hirayama M, Seino Y (2011). Efficacy and safety of alogliptin added to pioglitazone in Japanese patients with type 2 diabetes: a randomized, double-blind, placebo-controlled trial with an open-label long-term extension study. Diabetes Obes Metab.

[64] Kashiwagi A, Kadowaki T, Tajima N, Nonaka K, Taniguchi T, Nishii M, Ferreira JCA, Amatruda JM. Sitagliptin added to treatment with ongoing pioglitazone for up to 52weeks improves glycemic control in Japanese patients with type 2 diabetes. J Diabetes Investig. 2011;2:381–90. 10.1111/j.2040-1124.2011.00120.x PMC4019307 24843518

[65] Seino Y, Fujita T, Hiroi S, Hirayama M, Kaku K (2011). Alogliptin plus voglibose in Japanese patients with type 2 diabetes: a randomized, double-blind, placebo-controlled trial with an open-label, long-term extension. Curr Med Res Opin.

[66] Kawamori R, Inagaki N, Araki E, Watada H, Hayashi N, Horie Y, Sarashina A, Gong Y, von Eynatten M, Woerle HJ, Dugi KA (2012). Linagliptin monotherapy provides superior glycaemic control versus placebo or voglibose with comparable safety in Japanese patients with type 2 diabetes: a randomized, placebo and active comparator-controlled, double-blind study. Diabetes Obes Metab.

[67] Seino Y, Miyata Y, Hiroi S, Hirayama M, Kaku K (2012). Efficacy and safety of alogliptin added to metformin in Japanese patients with type 2 diabetes: a randomized, double-blind, placebo-controlled trial with an open-label, long-term extension study. Diabetes Obes Metab.

[68] Seino Y, Hiroi S, Hirayama M, Kaku K (2012). Efficacy and safety of alogliptin added to sulfonylurea in Japanese patients with type 2 diabetes: A randomized, double-blind, placebo-controlled trial with an open-label, long-term extension study. J Diabetes Investig.

[69] Kadowaki T, Tajima N, Odawara M, Nishii M, Taniguchi T, Ferreira JCA (2013). Addition of sitagliptin to ongoing metformin monotherapy improves glycemic control in Japanese patients with type 2 diabetes over 52 weeks. J Diabetes Investig.

[70] Kaku K, Mori M, Kanoo T, Katou M, Seino Y (2014). Efficacy and safety of alogliptin added to insulin in Japanese patients with type 2 diabetes: a randomized, double-blind, 12-week, placebo-controlled trial followed by an open-label, long-term extension phase. Expert Opin Pharmacother.

[71] Odawara M, Hamada I, Suzuki M (2014). Efficacy and Safety of Vildagliptin as Add-on to Metformin in Japanese Patients with Type 2 Diabetes Mellitus. Diabetes Ther.

[72] Hirose T, Suzuki M, Tsumiyama I (2015). Efficacy and Safety of Vildagliptin as an Add-on to Insulin with or without Metformin in Japanese Patients with Type 2 Diabetes Mellitus: A 12-week, Double-Blind, Randomized Study. Diabetes Ther.

[73] Tajima N, Kadowaki T, Okamoto T, Sato A, Okuyama K, Minamide T, Arjona Ferreira JC (2013). Sitagliptin added to voglibose monotherapy improves glycemic control in patients with type 2 diabetes. J Diabetes Investig.

[74] Kadowaki T, Tajima N, Odawara M, Minamide T, Kawashima M, Yanagida D, Okamoto T, Ferreira JCA (2013). Efficacy and safety of sitagliptin add-on therapy in Japanese patients with type 2 diabetes on insulin monotherapy. Diabetol Int.

[75] Tajima N, Kadowaki T, Odawara M, Nishii M, Taniguchi T, Ferreira JCA (2011). Addition of sitagliptin to ongoing glimepiride therapy in Japanese patients with type 2 diabetes over 52 weeks leads to improved glycemic control. Diabetol Int.

[76] World Health Organization. World Health Statistics 2015. 2015.

[77] Xie W, Zhao Y, Zhang Y (2011). Traditional Chinese Medicines in Treatment of Patients with Type 2 Diabetes Mellitus. Evid Based Complement Alternat Med.

